# AI and professional liability assessment in healthcare. A revolution in legal medicine?

**DOI:** 10.3389/fmed.2023.1337335

**Published:** 2024-01-08

**Authors:** Claudio Terranova, Clara Cestonaro, Ludovico Fava, Alessandro Cinquetti

**Affiliations:** Legal Medicine and Toxicology, Department of Cardiac, Thoracic, Vascular Sciences and Public Health, University of Padua, Padua, Italy

**Keywords:** artificial intelligence, machine learning, legal medicine, professional liability, tort law, causal relationship

## Abstract

The adoption of advanced artificial intelligence (AI) systems in healthcare is transforming the healthcare-delivery landscape. Artificial intelligence may enhance patient safety and improve healthcare outcomes, but it presents notable ethical and legal dilemmas. Moreover, as AI streamlines the analysis of the multitude of factors relevant to malpractice claims, including informed consent, adherence to standards of care, and causation, the evaluation of professional liability might also benefit from its use. Beginning with an analysis of the basic steps in assessing professional liability, this article examines the potential new medical-legal issues that an expert witness may encounter when analyzing malpractice cases and the potential integration of AI in this context. These changes related to the use of integrated AI, will necessitate efforts on the part of judges, experts, and clinicians, and may require new legislative regulations. A new expert witness will be likely necessary in the evaluation of professional liability cases. On the one hand, artificial intelligence will support the expert witness; however, on the other hand, it will introduce specific elements into the activities of healthcare workers. These elements will necessitate an expert witness with a specialized cultural background. Examining the steps of professional liability assessment indicates that the likely path for AI in legal medicine involves its role as a collaborative and integrated tool. The combination of AI with human judgment in these assessments can enhance comprehensiveness and fairness. However, it is imperative to adopt a cautious and balanced approach to prevent complete automation in this field.

## 1 Introduction

Artificial intelligence (AI) has multiple applications in the medical-surgical context, both in scientific research and clinical care. Artificial intelligence has been and is used, for example, for the following purposes: (1) the diagnostic interpretation of images in ophthalmology (1), dermatology (2), gastroenterology (3), anatomic pathology (4), and radiology (5); (2) the interpretation of signals derived from electronic devices (6) and molecular data (genetics, tumor markers, protein structures, and medical records with medical history collection) (7); (3) the development of vaccines and drugs (8); (4) the prediction of access volume for healthcare facilities, the risk of complications in hospitalized patients, potential hospitalization, potentially serious complications, and prognosis (9–11); (5) the more precise classification of diseases (12); and (6) robotic surgery (13, 14).

Although AI promises to improve the quality of care and patient safety, potential adverse events attributable to errors can still occur (15). The occurrence of errors in the context of healthcare professionals using AI or even, in the future, adverse events attributable to autonomous AI applied in the healthcare field will need to be evaluated differently from today and likely require judges, lawyers, and expert witnesses to have new skills (16, 17).

Although the use of AI in the forensic medical field for assessing professional responsibility has not been developed, AI’s application has already been expanded within the legal context, and discussions about its potential use as evidence in civil and criminal cases have taken place (18–20).

In view of the spread of medical litigation and the information presented above, it is worthwhile to delve into the current steps involved in the assessment of medical-professional liability cases and examine how AI can be integrated into these steps, as is already happening in specific contexts. This analysis will explore both how AI impacts the behavior of healthcare professionals and how it can assist expert witnesses in evaluating such cases. The authors will, therefore, first explain the differences between autonomous AI and AI that is integrated with the activities of healthcare professionals, describe the steps required today to assess a case involving professional liability, and then analyze how AI influences the process of evaluating the individual phases of professional-liability analysis.

## 2 Autonomous AI and working together with the AI

Autonomous AI and integrated AI in clinical healthcare represent distinct approaches to the use of AI in medical settings (21). The term “autonomous AI” refers to AI systems that can operate independently and make decisions without human intervention. On the other hand, integrated AI plays a supportive role by combining AI insights with human expertise (22). Currently, autonomous AI is not sufficiently advanced or trusted for full clinical application, while integrated AI is gaining increasing acceptance (23). For example, AI systems can now analyze medical images to suggest potential diagnoses (24) but physicians still review the AI output to make final diagnostic and treatment decisions (25).

In considering the concepts of “Human in the loop” and “Human out of the loop,” which describe the processes enabling the level of human involvement in decision-making and collaboration between humans and machines (26, 27), it becomes evident that enhancing human interaction and control is paramount for the responsible and effective use of AI. The Human-in-the-loop approach allows for the modification of machine learning (ML) methods’ results by incorporating human skills and expertise, enabling human interaction at every step of the ML process (28). These processes are widely acknowledged for improving medical workflows and enhancing patient safety. For instance, in the context of coma prognosis, a model has been studied that operates through a response loop. In this loop, the human’s intention is derived by collecting biological signals and context data, and the decision is then interpreted into a human-recognizable system action, completing the loop (29).

The specific design of interfaces emerges as a key factor in achieving the goal of enhancing human interaction and control. Tailoring user interfaces to facilitate transparent communication between humans and AI systems is crucial for better understanding and interpretation of AI-generated insights (30). Intuitive and user-friendly interfaces could enhance the understanding of a logarithmic decision with a high level of causal understanding, empowering individuals to effectively engage with and influence the decision-making process (31). Moreover, the incorporation of feedback mechanisms and alerts in interfaces can keep humans informed about AI-generated decisions, enabling them to intervene when necessary. This iterative feedback loop promotes a collaborative relationship between humans and AI, leveraging the strengths of both for optimal outcomes (32).

Considering the above, it seems unlikely that AI will completely replace human clinicians in the near term (33). While AI is becoming more capable, the practice of medicine involves intricate reasoning, interpersonal skills, and ethical considerations that AI currently lacks (34). Nevertheless, AI will play an increasingly integral role in clinical practice as an augmenting technology (35). The most probable trajectory involves physicians leveraging AI as a collaborative tool with which to enhance their abilities (36, 37).

This review will focus on the medico-legal aspects of integrated AI in clinical healthcare.

## 3 Description of the literature search

In June 2023, one of the authors (CT) conducted a comprehensive literature review by searching MEDLINE/PubMed. Temporal constraints were applied, limiting the scope to articles published within the last 5 years (2018–2023). Only English publications in full text were deemed suitable for inclusion in the study. The search employed the following phrases: “artificial intelligence medical malpractice,” “artificial intelligence legal medicine,” “artificial intelligence professional liability assessment,” and “artificial intelligence informed consent.”

The articles were meticulously reviewed by CC and the other authors, with a specific focus on elements pertaining to legal medicine and professional liability assessment. Additionally, relevant articles cited within the analyzed papers were taken into consideration, particularly those addressing key aspects of professional liability assessment using AI, including informed consent, damage objectivation, conduct evaluation, and causal relationship assessment.

## 4 Medical malpractice liability assessment

While there may be variations in civil and tort law across countries (38), there is a consensus regarding assessing medical malpractice and compensation for damages. This consensus establishes the presence of proven civil liability through an error that is causally linked to the patient suffering harm (5, 39). First, to assess professional liability, it is necessary to demonstrate the existence of patient harm due to medical malpractice. This harm can manifest as the transition from a healthy condition to a disease, result in death, or exacerbate a pre-existing condition (39). It represents a physical or psychological injury suffered by the plaintiff under tort and/or civil law (38, 40).

Demonstrating harm requires collecting clinical documentation, conducting a direct examination of the patient, and investigating the clinical situation through additional diagnostic tests (38). The reconstruction of the physiopathological course, which encompasses the actual sequence of events that occurred, is part of this phase. When evaluating a professional-liability case, it is also required to analyze the collection of informed consent from the patient.

Errors on the part of the healthcare professional are then identified to establish professional-malpractice liability. An error occurs when the physician deviates from the standard of care. The standard of care is typically determined by comparing the physician’s conduct to that of a competent physician with a similar level of specialization and available resources (5, 15). Guidelines, consensus documents, and evidence-based publications should guide the actions of a competent physician.

The last step in the analysis of a professional-liability case is the evaluation of the causal link between the error on the part of the physician and the event (damage) involving the patient (38).

### 4.1 Damage identification, reconstruction of physiopathological pathways, and AI

The initial stage of assessing professional-liability cases involves the objective determination of the harm incurred and the subsequent reconstruction of the pathophysiological events that transpired. Artificial intelligence possesses the ability to thoroughly analyze extensive datasets derived from medical records and scientific literature, facilitating the identification of pathophysiological patterns that may elude human observation. It can also analyze all the contributing factors leading to the causation of damage.

The objectification of damage can be carried out by employing AI, with the specific approach varying depending on the medical specialization relevant to the professional-liability case at hand. One of the techniques for objectifying damage includes the application of AI in diagnostic classification, as previously mentioned. In other fields, such as the psychiatric field, AI’s contribution could be more limited, as dictated by the subject, in terms of the objectivization of the damage.

### 4.2 Informed consent and AI

Valid informed consent from patients is crucial to healthcare professionals carrying out medical treatments legally and ethically (41). For consent to be genuinely informed and competent, the patient must be able to willingly receive and comprehend the relevant information; process the details; evaluate the situation and consequences; understand the benefits, risks, and alternatives; and communicate their decision (42). Thus, the information presented to patients to obtain valid consent must be clear, comprehensible, tailored to their level of understanding, and provided using language suited to their background (43).

Performing any medical procedure without acquiring valid consent is considered unethical and illegal in many places, even if no harm occurs, potentially leading to malpractice lawsuits and liability (44). When a patient is harmed, a lack of informed consent further weakens the physician’s legal position (45). Information is based on communication with patients, which may be influenced by physiological and pathological conditions. Age, diseases, and medications may alter the capacities of patients. The elderly and children are populations with specific needs regarding informed consent. Truly informed consent is particularly difficult in elderly patients because of their physical conditions (46), their medical conditions (47), the effects of medications (48), as well as their attitudes of passive acceptance rather than active involvement in their care (49).

Although the responsibility for informed consent primarily lies with the healthcare professional who frames the informed consent within the doctor-patient relationship, the use of AI systems in healthcare also presents challenges related to informed consent, particularly concerning vulnerable populations.

The accuracy of information may be inadvertently influenced by AI if the system provides erroneous recommendations that lead to harm for patients. This can result from biases in the representativeness of data used to train an algorithm leading to poor performances for certain patient populations (50). This could suggest the necessity of tailoring informed consent differently for these groups. Additional factors contributing to this problem include poor design choices and healthcare professionals’ failure to correctly interpret the AI system’s information (14). Informed consent may also be compromised due to the complexity of advanced statistical and machine-learning techniques, which are not easily explained to patients. The inner workings of the AI system can function as a “black box,” making it difficult to precisely describe its operations (51). Patients, especially those with specific difficulties or unrealistic expectations regarding the accuracy and objectivity of AI, may give consent that is not fully conscious and unambiguous.

Adhering to the principle of transparency, any known biases or limitations inherent in healthcare-based AI systems should be clearly communicated (52). However, it should also be questioned whether or not physician would be able to assess whether the AI system has been trained on a representative dataset of a particular patient population (53).

Lastly, the information provided by AI may shift the responsibility for any adverse events onto patients (16).

In summary, when evaluating the medical-legal aspects of informed consent, informational deficiencies (Table 1) may be attributed to the following:

**TABLE 1:** Informed consent and liability.

Subject liable	Reason
Physician	Failure to comprehend the information provided by the AI, limitations of the AI not being communicated to the patient, lack of consideration of the patient’s limited understanding for physiological or pathological reasons.
Patient	Underestimation of the recommendations provided by AI.
Producers, programmers, developers, with potential shared responsibility on the part of the purchasing company.	Erroneous recommendations provided by AI to the physician.

(1) Healthcare professionals who fail to comprehend the information provided by the AI and do not communicate the limitations of the AI to the patient or do not consider the patient’s limited understanding for physiological or pathological reasons.

(2) Patients who, even after being adequately informed, underestimate the recommendations provided by the AI system, for example, in the monitoring of a chronic condition.

(3) Artificial intelligence systems that have not been adequately trained by their developers or are affected by biases.

### 4.3 Analysis of the conduct of the physician and AI

A physician’s conduct is deemed appropriate when it aligns with the standard of care expected from a skilled physician practicing within the same medical specialty while making use of the available resources (15). The examination of a physician’s actions in a malpractice case by employing AI, with the capacity to analyze extensive medical data more efficiently and consistently than humans (54), will encompass an assessment of the various types of errors that may occur when a physician integrates AI into their practice (Table 2) (17, 40). Before specifically discussing the various types of errors that may be encountered when analyzing the conduct of a healthcare professional using artificial intelligence systems, it is necessary to clarify the concept of bias in the field of artificial intelligence and distinguish it from error. Biases can affect every stage of AI model development, including data collection (e.g., generation bias), data annotation (e.g., missing data bias), model development (e.g., training data bias), model deployment (e.g., user interaction bias), model evaluation (e.g., statistical bias) (55). The identification of sources of bias in AI algorithms is often impossible, so that a legal framework has been advocated that balances interests, responsibilities and liability risks among stakeholders, and ensures the reduction of biases in the design process before the release on the market (56).

**TABLE 2:** Alternative sources of errors in the conduct of the physician.

Type of error	Responsibility when damage is present and a causal relationship between error and damage is demonstrated
Error by healthcare professionals independently of AI’s role	Physician
Incorrect AI recommendations	Trainers and/or programmers, with potential shared responsibility on the part of the purchasing company.
Failure of the device.	Producers, with potential shared responsibility on the part of the purchasing company.
Failure to utilize AI when possible and necessary	Physician and/or the healthcare facility.
Failure when using AI and interpreting its results	Physician and/or the healthcare facility.

#### 4.3.1 Error by healthcare professionals independent of AI’s role

This type of error is classically associated with practices that are discordant with evidence-based medicine and unrelated to AI (57). They are the hypothetical responsibility of the physician.

#### 4.3.2 Incorrect AI recommendation

Inadequate training data or poor design choices with errors, can suggest incorrect conduct on the part of the physician, with harmful consequences (16, 58). In the clinical field, data quality is more important than the algorithm and often represents the main cause of incorrect indications (59). Insufficient data or overly complex algorithms can lead to overfitting, in which predictions are valid for a dataset but may prove unreliable given additional data (16, 60, 61). The demand for extensive data, often referred to as “data hungriness” (62), poses medico-legal challenges, as single institutions may lack sufficient data for reliable predictions (16). Finally, data cleansing can enhance data usability in the context of intelligence, but it must be implemented carefully to avoid introducing another source of errors (59, 63). This is the hypothetical responsibility of trainers and programmers, with potential shared responsibility on the part of the purchasing company, considering the limitations in the assessment related to the concepts of bias and error.

#### 4.3.3 Failure of the device

Hardware failures may contribute to errors (16, 58). They are the hypothetical responsibility of producers, with potential shared responsibility on the part of the purchasing company.

#### 4.3.4 Failure to utilize AI when possible and necessary

Misconduct arising from the failure to use AI when necessary, may be attributed to inadequate training in AI utilization on the part of the worker. Insufficient training can be caused by either the worker or the healthcare facility where they are employed. The choice of the most appropriate AI technology for the patient’s situation can represent a further source of error. The liability for such wrongful conduct will depend on the prevailing legislation in a given country and may require mandatory updates and training (64). It is the hypothetical responsibility of the physician and/or healthcare facility.

#### 4.3.5 Failure when using AI and interpreting the results of AI

Errors in data input and interpretation are another potential source of error. A physician could improperly evaluate the results provided by AI without considering the possibility of errors. Automation bias occurs when the physician passively accepts AI outputs that are wrong due to an operating error or training on wrong data (16, 17). Also, in these cases, a contributing factor may be the training of the worker. This is the hypothetical responsibility of the physician and/or healthcare facility.

Using AI when evaluating such conduct certainly allows the analysis of extensive medical data and guidelines more efficiently and consistently than humans would, but AI lacks nuanced human judgment (65). Medicine involves complex decision-making with imperfect information. While AI can identify patterns in data, it struggles to account for unique patient circumstances and subtle factors that justify deviations from guidelines (66). Consequently, AI assessments could over-rely on retrospective guidelines rather than evaluating the real-time context physicians face. This could lead to unfair conclusions about reasonable conduct. Additionally, biased data and algorithms could negatively impact the analysis of physician actions involving marginalized patient populations (67).

### 4.4 Causal relationship and AI

Medical malpractice occurs when a healthcare provider deviates from the accepted standard of care and causes harm to a patient. Establishing causation is essential in medical malpractice cases to show that the provider’s negligence directly caused the patient’s injury. However, determining causation can be complex, especially with multiple factors being involved in treatment and health outcomes.

Artificial intelligence and machine-learning tools have the potential to help establish causal relationships in medical-malpractice cases. These tools can analyze large datasets derived from medical records, treatment guidelines, and the scientific literature to identify statistically significant associations between interventions and outcomes (68). For example, an AI system could review thousands of cases involving a particular drug or procedure to determine whether it is linked to higher complication rates after controlling for other clinical factors (69). However, AI has limitations when determining causation. First, AI systems can operate as “black boxes,” making it a significant technical challenge to provide an explanation for their predictions (7, 70). When utilizing AI for assessing causal correlations, it is essential to consider the model’s accuracy in providing accurate predictions. This accuracy assessment should encompass the availability of reproducible studies (7).

Correlation does not necessarily imply causation, so simply because two factors are associated does not mean one caused the other. The evaluation of causation must be contextualized on a case-by-case basis through the determination of the medico-legal criteria, including universal and statistical laws, as well as the criterion of rational credibility (38). On one hand, AI could identify the most consistent scientific laws and guidelines; on the other hand, rational credibility relies on the judgment of a professional, who adapts scientific laws to clinical reality by considering all the variables involved. This becomes even more complex in the analysis of omissive medical errors, in which it is necessary to reconstruct the hypothetical alternative clinical course to define the causation of the injury. Moreover, the most complex professional-liability cases often involve the opinions of different physicians with expertise in the field, bringing together this expertise to achieve the highest degree of credibility. Artificial intelligence systems may find spurious correlations that do not reflect true causal mechanisms (71).

Human expertise is still required to contextualize AI insights and make sound judgments on causation that consider complex real-world clinical scenarios.

Overall, AI can be a useful tool for finding patterns in data to provide evidence for or against causation arguments in medical-malpractice cases (67). However, human analysis and discretion are still essential to determine whether negligence was the most probable cause of a patient’s injury (64). Artificial intelligence alone cannot definitively prove causation but should complement human evaluation (72). Handled responsibly, these technologies could improve the accuracy of determinations regarding liability and standards of care.

## 5 Time for a new expert witness in liability cases

The integration of AI into assessing professional liability is likely to necessitate a new type of expert witness. The qualifications to evaluate AI systems, the analysis of a process, the speed and scale of case analysis, communication skills, and perceived objectivity represent the differences between a traditional expert witness and an expert witness leveraging AI in medical-malpractice analysis. A new technical consultant must also be familiar with emerging legislation pertaining to artificial intelligence, especially in the context of healthcare applications. For instance, the European Union’s Regulation on *In vitro* Diagnostic Medical Devices (IVDR) exemplifies legislative efforts to regulate artificial intelligence in healthcare, with potential professional responsibility implications. This regulation introduces AI-based analysis software in medical diagnostics for decision support. However, it mandates that developers design these tools in accordance with the state of the art, a concept encompassing safety, verification, and validation of the underlying data. Essentially, the legislation requires explainable and understandable artificial intelligence systems to enable healthcare professionals to make responsible clinical decisions as required by law (73).

As machine-learning algorithms can be applied to analyze conduct against standards of care, the validity, biases, and limitations of these models will require expert examination (74). Traditional expert witnesses may lack the skills needed to critically assess AI systems. Knowledge gaps regarding data preprocessing, model selection, training approaches, and algorithmic bias checks could hamper the evaluation of AI reliability and fairness (75). Without AI fluency, witnesses may struggle to explain inherent uncertainties or be vulnerable to problematic assumptions (76). The “new” expert witness could review and compare many more cases using AI automation; when backed by data, they could be perceived as more impartial, and fact based.

Ideal candidates should combine domain knowledge in their professional field with AI expertise to translate technical details for legal professionals (15). They can opine on the comprehensibility, generalizability, and potential discrimination of AI systems for liability analysis (65). Through testimony, they can provide an essential perspective on the appropriate role of AI vs. human judgment.

In summary, the introduction of AI to assist in determining professional liability necessitates impartial experts who can credibly evaluate the technology. Developing a cadre of qualified AI expert witnesses will be key to ensuring due process as algorithmic tools are adopted in legal realms. The optimal approach integrates the best of both worlds in the form of AI augmentation without full automation. However, the shifting balance toward data-driven analysis marks a potential evolution in expert practice.

## 6 Conclusion

The integration of AI into the assessment of professional liability represents a major evolution in legal medicine, albeit one with growing pains. As described, AI streamlines the analysis of the multitude of factors relevant to malpractice claims, including informed consent, standard-of-care adherence, and causation. However, solely relying on AI autonomy at this stage poses concerning risks regarding biased algorithms, a lack of nuance, and overall credibility. Therefore, the prudent path forward entails integrating AI tools with human experts’ input. The presence of bias is of significant relevance in determining professional liability, which is influenced by the legal framework. In fault-based frameworks, the focus is usually on demonstrating negligence or misconduct, consistent with the conventional view of medical malpractice. In these instances, the existence of bias can impede the determination of culpability. Conversely, no-fault liability models prioritize compensating victims without requiring proof of fault. Per some scholars, this methodology may be imperative if AI is deployed in healthcare (77).

Our analysis suggests that it’s time to consider a new expert witness for liability cases. On the one hand, artificial intelligence will support the expert witness; however, on the other hand, it will introduce specific elements into the activities of healthcare workers. These elements will necessitate an expert witness with a specialized cultural background.

By combining AI’s high-volume data processing with human judgment, oversight, and explanation, professional liability assessments could become more comprehensive and equitable. Of course, the ideal balance of responsibilities between humans and machines remains unclear. We must take care to ensure AI amplification, not automation, in this high-stakes domain. If undertaken judiciously, AI integration offers legal medicine an unprecedented opportunity to improve the consistency, efficiency, and fairness of professional-liability determinations.

## Author contributions

CT: Conceptualization, Writing – original draft, Writing – review and editing. CC: Supervision, Writing – review and editing. LF: Writing – review and editing. AC: Writing – review and editing.
